# Preventive Effect of Halofuginone on Concanavalin A-Induced Liver Fibrosis

**DOI:** 10.1371/journal.pone.0082232

**Published:** 2013-12-16

**Authors:** Jie Liang, Bei Zhang, Ruo-wu Shen, Jia-Bao Liu, Mei-hua Gao, Ying Li, Yuan-Yuan Li, Wen Zhang

**Affiliations:** 1 Department of Immunology, Medical College of Qingdao University, Qingdao, China; 2 Department of Anatomy, Medical College of Qingdao University, Qingdao, China; 3 Department of Radiology, Affiliated Hospital of Qingdao University Medical College, Qingdao, China; French National Centre for Scientific Research, France

## Abstract

Halofuginone (HF) is an active component of extracts derived from the plant alkaloid febrifugine and has shown therapeutic promise in animal models of fibrotic disease. Our main objectives were to clarify the suppressive effect of HF on concanavalin A (ConA)-induced liver fibrosis. ConA injection into the tail vein caused a great increase in the serum aspartate aminotransferase (AST) and alanine aminotransferase (ALT) levels, while orally administration of HF significantly decreased the levels of the transaminases. In addition, the levels of hyaluronic acid (HA), procollagen III (PCIII) and TGF-β1 in the serum and collagen I, α-SMA, tissue inhibitors of metalloproteinase 2 (TIMP2) and Smad3 in the liver tissue were significantly lowered with the treatment of HF. Histological examination also demonstrated that HF significantly reduced the severity of liver fibrosis. Since ConA-induced liver fibrosis is caused by the repeated activation of T cells, immunomodulatory substances might be responsible for the suppressive effect of HF. We found that the production of nuclear factor (NF)-kB in the serum was increased in ConA-treated group, while decreased significantly with the treatment of HF. The changes of inflammatory cytokines tumor necrosis factor (TNF-α), IL-6 and IL-1β in the serum followed the same rhythm. All together, our findings indicate that orally administration HF (10ppm) would attenuate the liver fibrosis by suppressing the synthesis of collagen I and inflammation-mediated liver injury.

## Introduction

In any chronic liver disease (CLDs), whatever the aetiology, reiteration of liver injury results in persisting inflammation and progressive fibrogenesis. The normal liver structure may ultimately develop into overt cirrhosis after distorted by scar tissue. In these progress, tissue injury recruits inflammatory cells and activates hepatic stellate cells (HSC) which is the major source of ECM proteins in the injured liver[1] and of many of the metalloproteinases (MMPs) and their inhibitors (TIMPs)[2]. MMPs are a family of highly homologous metal-dependent endopeptidases that can cleave the majority of constituents of the extracellular matrix such as collagen, fibronectin, laminin and elastin[3]. MMPs are inhibited by endogenous tissue inhibitor of metalloproteinases (TIMPs)[4]. Chronic liver injury and activation of HSCs lead to the upregulation of TIMPs and growth factor β-1 (TGF-β1) with the inhibition of MMP activity. The TIMPs activation thus stimulates collagen I synthesis and matrix proteins accumulation in the extracellular space[5]. At cellular levels, the perisinusoidal HSC has been extensively reported as a key effector of fibrogenesis[5,6]. Following hepatocyte injury, HSC differentiates into an “activated” myofibroblast-like phenotype[7]and contributes to fibrillar collagen formation, which plays an important role in controlling liver fibrosis[8,9]. Moreover, it increases until vascular structures are linked and the architecture of the liver is disrupted significantly [10,11]. Although numerous agents have been tried, the lack of specific inhibitors of ECM components in general and the lack of specific inhibitors of collagen type I in particular, limits the progress in the treatment of hepatic fibrosis.

Many cytokines can regulate fibrosis through stimulating proliferation after binding to specific receptors on fibroblasts, attracting inflammatory cells, enhancing collagen production and autocrine factors secretion, including transforming growth factor-β1 (TGF-β1), tumor necrosis factor (TNF-α), Interleukin( IL)-1β and IL-6. TGF-β1 is recognized as a “master switch” to induce fibrosis, as well as EMT and myofibroblast generation. The direct targets in TGF-β1 pathway, Smads (Smad2, and especially Smad3), were critical mediators in fibrogenesis and EMT[12,13]. IL-1β and TNF-α play similarity effects on fibroblasts[14]. IL-1, a ubiquitous and pleiotropic cytokine, particularly IL-1β, inhibits collagen production but it enhances fibroblast proliferation. Similarly, TNF-α, a primary immune and inflammatory regulator, stimulates fibroblast chemotaxis and proliferation meanwhile it inhibits collagen production[15,16]. IL-6, another inflammatory cytokine, which may affect differentiation of fibroblast to myofibroblast, plays an important role in fibrosis diseases[17,18]. 

NF-KB (nuclear factor kappa-light-chain-enhancer of activated B cells) is a family of transcription factors which plays a critical role in regulation of immunity and inflammation by stimulating the transcription of a wide range of cytokine-encoding genes, including TNF-α and IFN-γ. This family is composed of five related transcription factors (p50, p52, p65, c-Rel, and RelB), and they can form homo- and heterodimers[19]. The most important NF-kB dimmers are formed by p65 and p50 in NF-kB signaling pathway[20]. NF-kB mediated transcriptional activation plays a critical role in the HSC activation [21]. NF-kB activity can induce the expression and secretion of the various inflammatory cytokines and adhesion molecules, which play a major role in hepatic fibrosis [22,23]. Upon stimulation by inflammatory cytokines such as TNF-*α*, IkB is phosphorylated by I*KK* and degraded. NF-kB is then released and translocates to the nucleus from cytoplasm, and activates the transcription of its target genes[22]. Therefore, inhibition of NF-kB activity is considered as an underlying mechanism for anti-fibrosis[24,25].

For centuries, the roots of Dichroa Febrifuga, a saxifragaceous plant, have been used in china in the treatment of malarial fever. Febrifugine and its stereoisomere, isofebrifugine, were identified as the active antimalarial components[26]. Halofuginone (HF) [7-bromo-6-chloro-3-(3-hydroxy-2-piperidine)-2-oxopropyl-4(3H)-quinazoline] is one of the febrifugine analogous used world-wide for almost 20 years in commercial poultry production to prevent coccidosis[27,28]. In addition, halofuginone has been found to attenuate collagen α 1 (I) gene expression and collagen production by murine ,avian and human skin fibroblasts derived from either scleroderma or chronic graft-versus-host disease(cGvHD) patients[29,30]. It can inhibit collagen α 1(I) gene expression in many models of fibrosis involving skin, liver, urethra, heart, surgical and traumatic adhesions[31,32]. This inhibitory effect was probably mediated by blocking TGF-β induced Smad3 activation [33]. TGF-β is a central regulator in chronic liver disease, being involved in all stages of the disease progression, from initial liver injury, inflammation, fibrosis, to cirrhosis and hepatocellular carcinoma at the end[27]. In addition, M. Leiba showed that halofuginone inhibited DTH response in mice, indicating suppression of T cell-mediated inflammation and pro-inflammatory cytokine production in vivo[34]. 

Concanavalin A (ConA)-induced liver injury is well accepted as a rat model of immune-mediated liver injury that resembles viral and autoimmune hepatitis in humans. The intravenous injection of ConA into rat can increases transaminase and activate T cells infiltrate in liver[35]. The activation of T cells by ConA results in increased levels of inflammatory cytokines, including TNF-α, IFN-γ and IL-6 [36]. In the present study, we assessed the preventive effect of HF on ConA-induced chronic liver fibrosis.

## Materials and Methods

### Ethics Statement

All the procedures and care administered to the animals have been approved by the Institutional Ethics Committee of Qingdao University Medical College, under a permit of animal use (SCXK40090007) in the Center of Experimental Animal and Animal Experiment at Qingdao, compliance with the Experimental Animal Regulations by the National Science and Technology Commission, China.

### Animals

Male Wistar rats (weighing 210-230 g) were supplied by the Experimental Animal and Animal Experiment Center of Qingdao, Shandong, China. They were housed in the animal facility with a 12 h light/dark cycle, the temperature was maintained at 22-23°C and relative humidity was 60%.

### Materials

ConA and HF were purchased from Sigma-Aldrich Co. (St Louis, MO, USA) and Jilan Technology Development Co.(Shanghai, China), respectively. NF-kB, TGF-β1, TNF-á, IL-6, IL-1β, HA and PC-III enzyme-linked immune sorbent assay(ELISA) kits and α-SMA, Collagen1, TIMP2, Smad3 polyclonal antibodies were from Solarbio Science &Technology Co. (Beijing , China) and Biosynthsis Biotechnology Co.(Beijing, China).

### Experimental design

**Figure 1:**
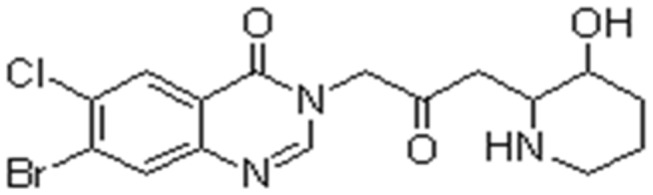
The chemical structure of halofuginone (HF).

HF (Figure 1), an alkaloid originally extracted from the plant Dichroa febrifuga was given in the diet at concentrations of 10 ppm. The animals were randomly distributed into three groups: group 1 (G1), rats received a weekly iv injection of 300ul PBS for 8 weeks (n =15); group 2 (G2), rats received a weekly iv injection of ConA (17.5mg/kg, in 300ul PBS) for 8 weeks (n =15); group 3 (G3), weekly iv injection of ConA (17.5mg/kg, in 300ul PBS) with 10ppm HF in diet for 8 weeks (n =15). During all experiments rats were maintained in individually ventilated cages under specific pathogen-free conditions. Animals were sacrificed on d57, blood was collected via cardiac puncture and serum was prepared by centrifugation at 2000g for 10 min and stored at -20°C. Livers were taken after perfusion with 4% paraformaldehyde. 

### Liver function and fibrosis index

The serum levels of alanine aminotransferase (ALT), aspartate aminotransferase (AST), total protein(TP) and the albumin (ALB) were measured with an auto-biochemical analyzer (Roche P800, Basle Switzerland). HA, PCIII were measured using ELISA micro-titer plates pre-coated with antibodies specific to HA or PCIII according to the instructions of the manufacturer. Each experiment was done in quadruplicate, and the results are expressed as the mean±SEM. 

### Cytokine Secretion

Sera aliquots collected from all rats were assayed for transforming growth factor-beta (TGF-β1) as a fibrogenesis-driving cytokine, the nuclear transcription factor NF-kB and the pro-inflammatory cytokine

Interleukin TNF-α, IL-6 and IL-1β as important signals in liver injury. All of them were assayed by enzyme-linked immune sorbent assay using ELISA micro-titer plates pre-coated with antibodies specific to NF-kB, TNF-α, IL-6 or IL-1β according to the instructions of the Manufacturer. Each experiment was done in quadruplicate, and the results are expressed as the mean±SEM. 

### Haematoxylin-eosin (HE) Staining

After rats were sacrificed, vessels were perfused with PBS, followed by 4% paraformaldehyde. Paraformaldehyde-fixed liver specimens were dehydrated in a graded alcohol series. Following xylene treatment, the specimens were embedded in paraffin blocks, cut into 4-μm thick sections, and placed on glass slides. The sections were then stained with hematoxylin and eosin (HE) according to standard procedures.

### Histopathological evaluation of liver

Liver fibrosis was assessed with Masson trichrome (MT) stain according to standard procedures. To describe and evaluate liver pathological changes, a pathologist who was blinded to the research design examined 10 different low-power fields of MT-stained sections (selected fields were in almost the same location) for each rat. In addition, the percentage of collagen calculated by a multimedia color image analysis system (Image-Pro Plus 6.0) was measured as a relative objective index (because a histological/fibrosis score that is evaluated by pathologists is susceptible to the ability and subjective judgment of the pathologist) to evaluate the degree of liver fibrosis. Each MT-stained section was examined at high-power fields (HPFs) (magnification ×400). Every field analyzed contained a granuloma, portal area, or a centrilobular vein. Fibrotic areas were scanned and summed by the software. The level of fibrosis was scored according to liver fibrosis semi-quantitative scoring system (SSS) method[37].

### Immunohistochemistry

Paraffin blocks were cut into 4-µm sections, deparaffinized in xylene, and rehydrated in graded ethanol solutions. Immunohistochemical SP method was used to detect the expressions of collagen I, α-SMA and Smad3. The tissue sections were first incubated a specific antibody (anti-α-SMA, anti-collagen I and anti-Smad3) overnight at 4 °C, washed in Phosphate Buffer Solution (PBS) for three times, and was then incubated with biotinylated secondary antibody (anti-immunoglobulin from the animal species from which the specific antibody was obtained) for 20 minutes at 37°C. After washed in PBS, the sections were incubated with avidin conjugated to HRP for 20min at 37°C, then colored with 3,3-diaminobenzidin (DAB). After counterstained with haematoxylin for 5 mins, tissue sections were washed and then dehydrated with ethanol from 70% to 80%, then 90%, 95%, 100%, followed by xylene (3X). Finally, the sections were covered with cover slips by neutral gums and observed by microscope. PV-6000 ploymer detection system was used to detect the expressions of TIMP2 in rat liver tissue according to the manufacturer’s instructions.

Image Pro plus 6.0 (Media Cybernetics, Bethesda, MD, USA) was used to quantify immune-staining results. This software was designed to select stained nuclei or cells based on color intensity and nuclear shape. Brown staining was considered positive. The chromatic area and the strength (light density values) of positive cells were calculated and represented as a percentage of total positively stained cells with integral light density values (integral optical density).

### Statistics

All statistical analyses in this study were carried out using Sigma-Plot 10 and SPSS 19.0, and results are expressed as the mean±SEM. A one-way analysis of variance (ANOVA) followed by the post-hoc Dunnett’s test was used to analyze multiple groups for evaluating statistical significance. A value of P<0.05 was considered significant.

## Results

### Treatment with HF decrease LBWR in ConA-treated rats

**Figure 2:**
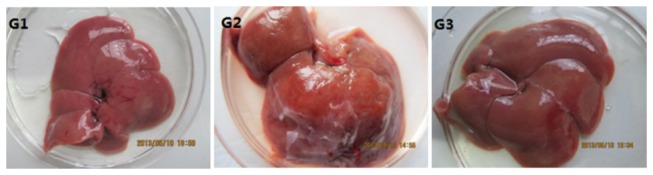
Macroscopic view of the liver from different groups. Macroscopic view of the liver from ConA-treated rats, showing a bigger volume and a rough surface with many nodules that developed on a liver with fibrosis, while it was similar to the normal and no obvious damage to the naked eye from ConA+HF treated rats.

There are 15 rats in G1, 10 and 13 left in G2 and G3 respectively when the animals were sacrificed after 8 weeks treatment. The body and liver weights were significant different among three groups. The liver-to-bodyweight ratio (LBWR) was significantly higher in G2 compared with that in G1, and simultaneous administration of halofuginone (8 weeks, 10 ppm) decreased the LBWR in G3 by 10.4% (Table 1), which was consistent with the macroscopic change of liver. In contrast to the livers in control group, a typical fibrosis appearance with increased volume and extensive nodular formation was present in the livers of ConA- treatment group, while the hepatic lesion were hardly observed under microscopy in ConA+HF group (Figure 2). Those results indicated that HF can decrease LBWR in ConA-induced liver fibrosis rats.

**Table 1:** Comparison of LBWR in three groups.

Group	n	LBWR
G1	15	4.53±0.0024
G2	10	5.42±0.0056^**^
G3	13	4.86±0.0031*^##^

There are significant difference in LBWR among this three groups. Data are presented as the mean ±SEM. Note: *p<0.05,**P<0.01 compared with G1, ^*#*^ p<0.05, *^##^* p<0.01 compared with G2.

### HF attenuates the impairment in liver function

**Figure 3:**
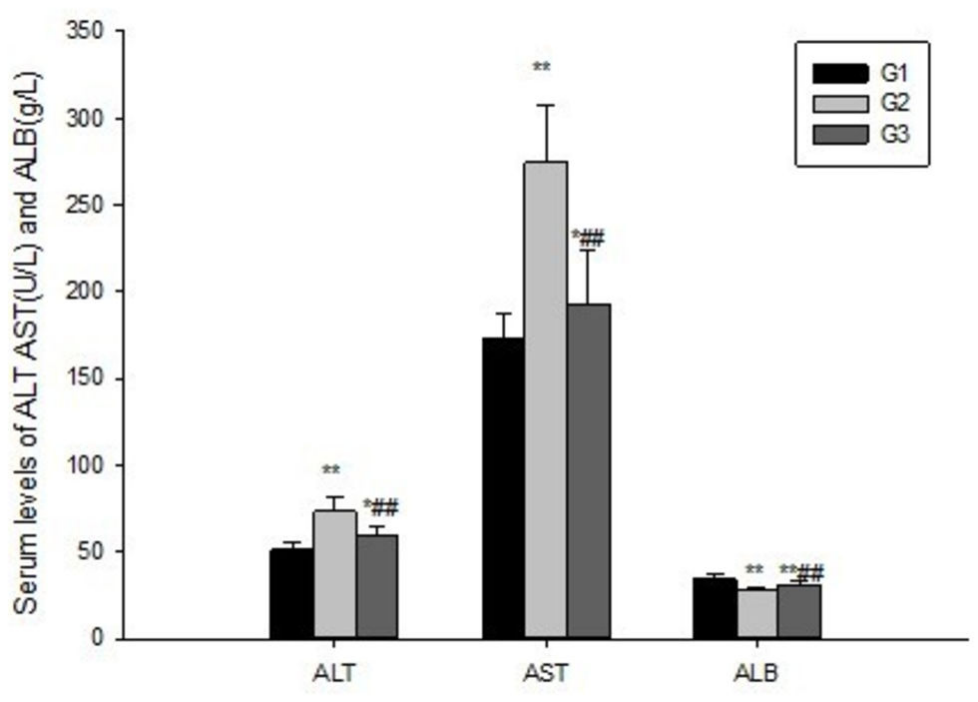
Halofuginone suppress liver injury induced by ConA. Blood was collected to determine the serum levels of AST, ALT and ALB. HF significantly decreased ALT, AST and increased ALB levels in serum. The values are the means ±SEM. *p<0.05, **p< 0.01 as compared to G1, ^#^ p<0.05, ^##^p<0.01 as compared to G2.

To investigate the effect of HF on ConA-induced liver injury, plasma ALT, AST and ALB levels were determined with an auto-biochemical analyzer. Preliminary studies revealed that the ALT, AST content were increased and albumin was decreased after repeated injections of ConA in a time-dependent manner, reaching a maximum and minimum at eight weeks (data not shown). ConA stimulation significantly increased plasma ALT(73.37±8.42U/L), AST(274.64±32.10U/L) and decreased ALB(27.58±1.87g/L) levels in G2 compared to G1, but simultaneous treatment with HF significantly lowered the level of ALT(59.29±5.63U/L), AST(193.02±30.83U/L) and increased the level of ALB(31.17±2.51 g/L) (Figure 3). Those results thus indicated that HF can attenuate the impairment of liver function induced by ConA injection.

### HF reduce fibrosis index

**Figure 4:**
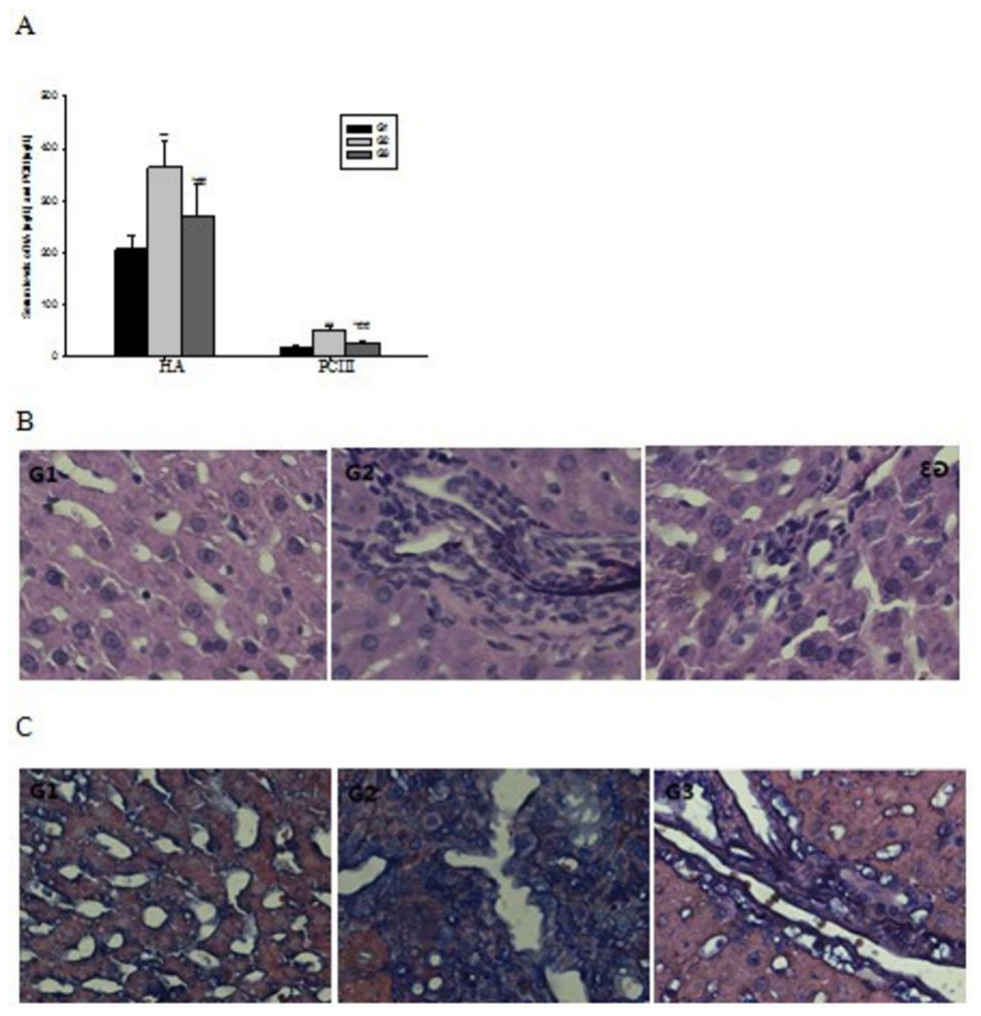
Halofuginone alleviate liver fibrosis level induced by ConA. (A)Treatment with halofuginone can markedly decreased the raised levels of HA and PCIII in serum induced by ConA. (B) H&E staining show enhanced collagenous fiber deposition expands in the portal area as well as in the bile duct in ConA-treated rats and the collagenous fiber attenuate obviously when there are halofuginone was added in diet. (C) MT staining shows collagen accumulation were the highest in ConA-treated rats, while it was decreased significantly with the treatment of halofuginone. Magnification for all photographs, ×400, the values are the means ±SEM. *p<0.05, **p< 0.01 as compared to G1, ^#^p<0.05, ^##^p<0.01 as compared to G2.

The levels of HA and PCIII, which have been shown as two liver fibrosis indexes, were detected by ELISA kits. Their production were significantly higher in G2 after ConA treatment for eight weeks. However, simultaneous administration of halofuginone (8 weeks, 10 ppm) decreased HA and PCIII production by 26% and 47% respectively, suggesting that HF can reduce the degree of liver fibrosis (Figure 4A).

### HF alleviates liver fibrosis level in ConA-treated rats

Weekly ConA injection for eight weeks resulted in advanced liver fibrosis, characterized by distorted tissue architecture with collagen bundles surrounding the lobules and large fibrous septa. Histopathologic findings of the rats liver tissues were shown in Figure 4B. In contrast to the normal foliages and cell structures seen in G1 liver tissue, HE staining displayed that the tissue architecture in G2 was distorted, with collagen bundles surrounding the lobules and a large number of inflammatory cell infiltrations together with hepatocellular necrosis. When compared with the G2, fibrous strips and inflammatory cell infiltration were markedly attenuated in the G3. The semi-quantitative scoring system (SSS) value for G3 was significantly lower than G2 (Table 2, P<0.05). 

**Table 2:** Fibrosis score of rats in different groups.

Group	N	Score
G1	15	0
G2	10	21.6±3.134^*^
G3	13	10.85±2.075*^##^

Haloluginone caused a significant reduction in the mean liver fibrosis score of the rats after 8 weeks of treatment. Data are presented as the mean ±SEM. Note: *p<0.05,**P<0.01 compared with G1, ^*#*^ p<0.05, *^##^* p<0.01 compared with G2.

The degree of fibrosis determined by MT staining of the liver sections from all the treated groups was shown in Figure 4C. Liver sections from normal rats appeared normal foliages without signs of collagen deposition. In contrast, liver sections from the G2 rats displayed increased deposition of collagen fibers surrounding the congested central vein, and a large number of inflammatory cell infiltration along with hepatocellular necrosis. Liver tissues from rats treated with HF showed mild collagen deposition and mild congestion around the central vein. The mean score of fibrosis in the G3 group was significantly lower than that of G2 (P<0.05). Measurement of the degree of fibrosis thus confirmed the previous findings that treatment with HF might protect the animals’ liver from development of fibrosis.

### HF supress the synthesis of Collagen 1, α-SMA and TIMP2

**Figure 5:**
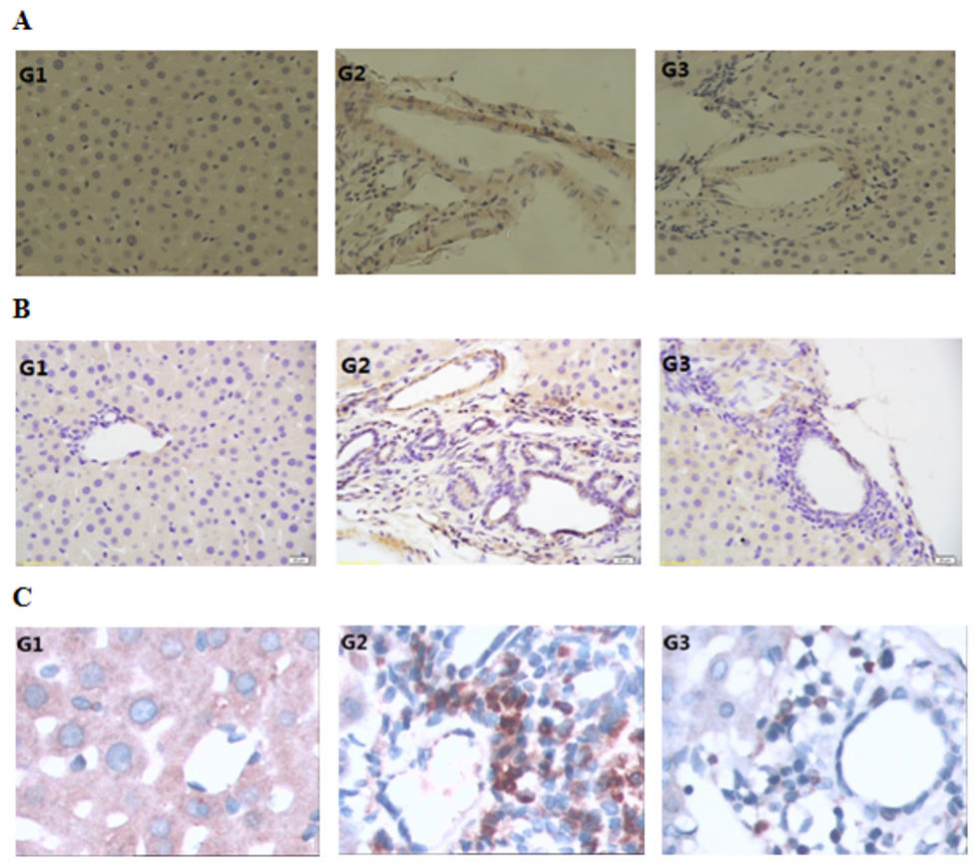
Halofuginone suppress the protein synthesis of Collagen 1, α-SMA and TIMP2. The expression of Collagen 1, α-SMA and TIMP2 were the highest in ConA-treated fibrosis rats, and their expressions were decreased significantly with the treatment of HF in G3. Magnification for collagen1 and α-SMA, ×200, for TIMP2, ×400.

In our present study, a remarkable collagen a 1(I) accumulation was observed in the rats from G2 group which received ConA treatment for eight weeks. In addition, TIMP-2 and stellate cells positive for α-SMA were also noted G2 group rats. HF-treated rats presented minimal levels of collagen1, α-SMA and TIMP2 expression throughout the experiment (Figure 5A-C). Neither α-SMA positive cells nor collagen and less TIMP-2 staining cells were observed in control animals. .

### HF down-regulates TGF-β1/Smad3 signaling pathway

**Figure 6:**
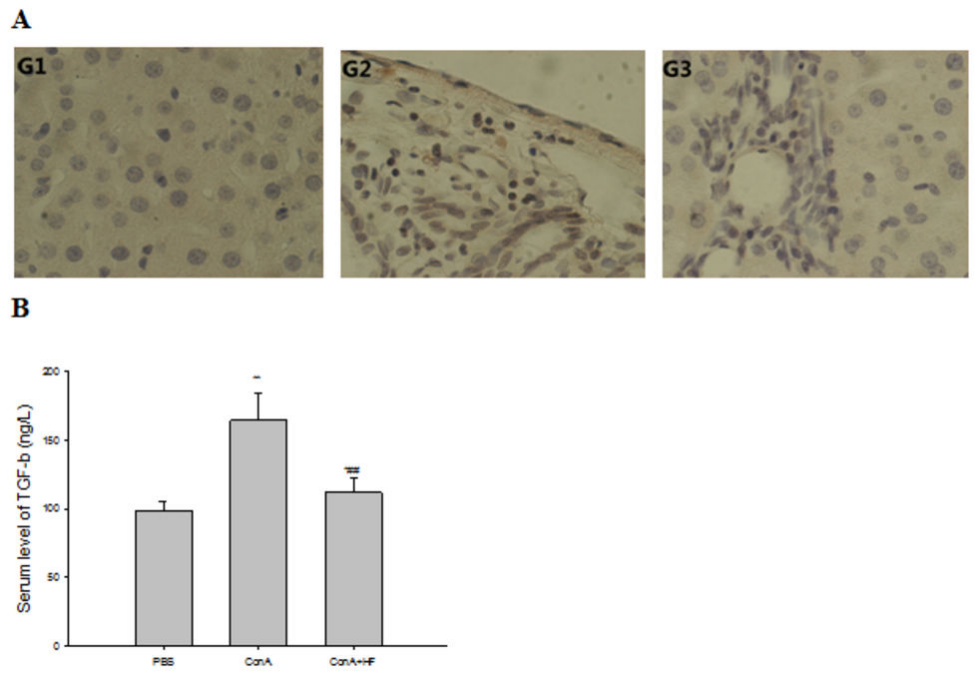
HF affects TGF-β1/Smad signaling pathway. TGF-β1 and Smad3 increased significantly in ConA-treated fibrosis rats, and HF attenuated the increased TGF-β1 and Smad3 markedly. The values are the means ±SEM. *p<0.05, **p< 0.01 as compared to G1, ^#^p<0.05, ^##^p<0.01 as compared to G2.

Since the TGF-β1/Smad3 signaling pathway has been shown to be involved in the regulation of fibrosis, and the expression of TGF-β1/Smad3 signaling pathway related protein collagen a 1(I), a-SMA and TIMP-2 have been measured by immunohistochemical methods. We further tested the expression of Smad3 in the liver tissue and the expression of TGF-β1 in the serum, in order to explore whether TGF-β1/Smad3 pathway was also affected with the treatment of HF. As shown Figure 6A, the expression of Smad3 was significantly increased in G2 rats treated with ConA for eight weeks, while its expression was obviously decreased after treatment with HF. The change of TGF-β1 expression in serum followed the similar tendency (Figure 6B). Overall, these results confirmed that HF inhibits the protein expression of type I collagen, TIMP2 and α-SMA in rats, which may be due to the down-regulation of TGF-β1/Smad3 signaling pathway.

### HF decrease inflammation cytokine secretion

**Figure 7:**
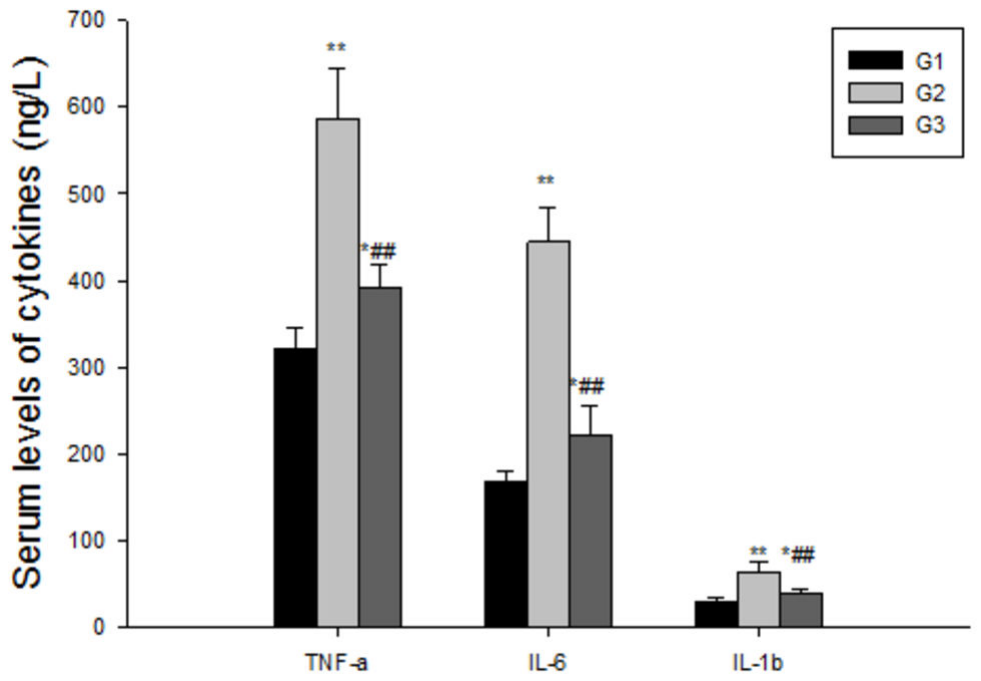
Supression of inflammatory cytokines secretion with the treatment of halofuginone. The serum levels of TNF-α, IL-6 and IL-1β were increased significantly with the treatment of ConA, while this three parameters were decreased abviously when there are halofuginone was added in diet. The values are the means ±SEM. *p<0.05, **p< 0.01 as compared to G1, ^#^p<0.05, ^##^p<0.01 as compared to G2.

G2 group rats expressed significantly higher levels of pro-inflammatory cytokines including TNF-α, IL-1β and IL-6, which have been shown to play important roles in the development of the fibrosis. However, simultaneous administration of HF resulted in remarkable decreases in the levels of all those three parameters, indicating that HF can decrease the secretion of inflammatory factors (Figure 7).

### HF reduce serum levels of NF-κB

**Figure 8:**
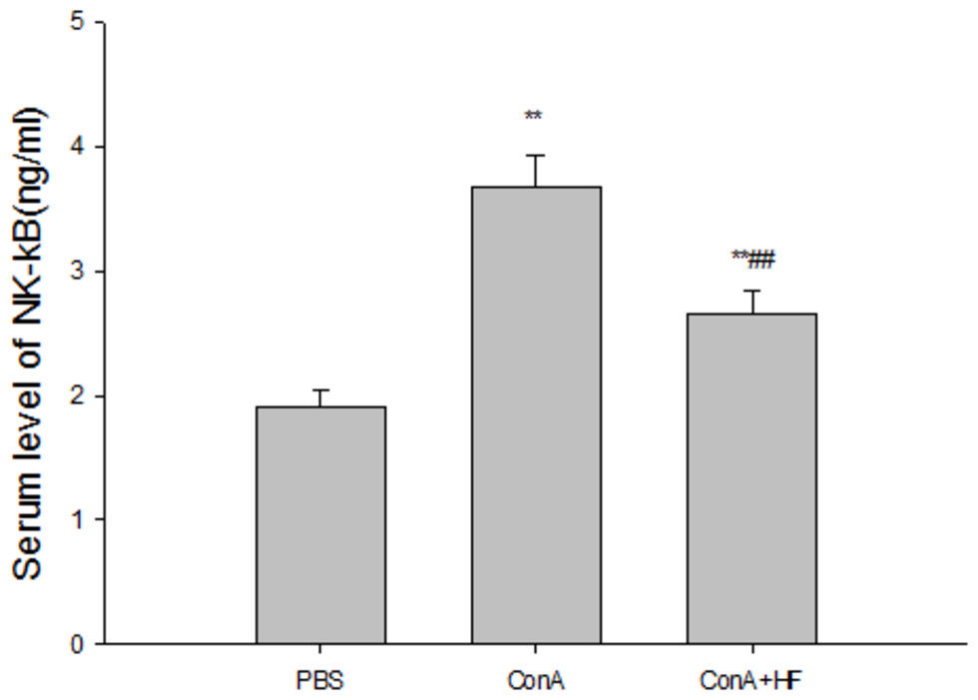
Halofuginone reduce the secretion of NF-kB. HF significantly surpress the increased serum NF-KB induced by ConA. The values are the means ±SEM *p<0.05, **p< 0.01 as compared to G1, ^#^p<0.05, ^##^p<0.01 as compared to G2.

The transcriptional factor NF-κB has been shown to be involved in the regulation of cytokine signaling and inflammation. We next examined the effects of HF on the level of NF-κB in the serum and found that the production of NF-κB in ConA-treated rats was significantly higher than that in the controls (Figure 8); however, treatment with HF suppressed NF-κB expression in the serum. Taking together, these data indicated that the anti-inflammation effects of HF treatment against ConA might be mediated by NF-κB signaling.

## Discussion

ConA-induced liver fibrosis, which was characterized by activation of the T cells, is considered more suitable for the study of liver fibrosis, because it resembles the liver fibrosis originated from viral and autoimmune hepatitis in humans. Repeated intravenous injection of ConA into rat increased the plasma transaminase (Figure 3) and fibrosis index level(Figure 4), meanwhile ConA injection activated T cells which could infiltrate the liver and cause the apoptosis and necrosis of hepatocytes eventually [38]. 

Our study showed here that HF played a hepatoprotective effect against ConA-induced liver fibrosis in rat, a widely used animal model to study the fibrosis induced by viral and autoimmune hepatitis [36]. Halofuginone, a well known inhibitor of collagen a1(I) gene expression, has recently been shown to prevent dimethylnitrosamine(DMN)[39] and thioacetamide-induced liver fibrosis[30]. Since collagen α1(I) is a major component in the body, it is of concern when one wants to treat patients systemically with an inhibitor of collagen like HF[36]. Moreover, HF treatment significantly reduced ECM components thereby improving overall liver architecture in established fibrosis. In our study, since the development of oral medications to help prevent liver injury is desirable[40], orally treatment with HF significantly improved liver histology(Figure 4B) and declined the high values of Collagen α1(I) protein(Figure 5A), and we found the decline in collagen deposition was accompanied by a reduction in numbers α-SMA positive cells(Figure 5B), pointing to the fact that HF may affect collagen levels by more than one mechanism. 

In liver fibrosis, there is an imbalance between excessive deposition and/or decreased removal in the extracellular matrix (ECM) with consequent scarring damage[41]. ECM is mainly controlled by matrix metalloproteinases (MMPs) and their inhibitors (TIMPs). MMPs belong to a group of proteolytic enzymes that are able to degrade the ECM, while TIMPs can inhibit the degradation of collagens. An increase in MMPs and TIMPs is commonly observed in fibrotic diseases. Park et al. reported that the imbalance of MMPs and TIMPs is the key factor of liver fibrogenesis[41]. In this study, we demonstrated that administration of HF inhibited the expression of TIMP2, thus reduced ECM deposition within the liver parenchyma and alleviated liver fibrosis. Therefore, downregulation of TIMP2 by HF treatment may contribute to the reduced fibrosis in the HF-treated rats.

Another mechanism by which HF could influence liver fibrosis is via the inhibition of TGF-β1 secretion. TGF-β1 is a profibrogenic cytokine in the liver, and has been shown to regulate multiple fundamental cellular processes, including cell growth, migration, adhesion, ECM deposition, and apoptosis [42-44]. Blocking TGF-β1 by adenovirus encoding a truncated type II receptor prevented the progression of fibrosis in DMN-injected animals[45,46]. The mechanism by which HF reduces fibrosis was recently elucidated in a mouse model for scleroderma (dermal fibrosis), where a low dose of halo blocked TGF-β mediated SMAD3 activation in fibroblasts[47]. Moreover, Xavier.et al, have recently demonstrated that halofuginone induced expression of the inhibitory Smad7 and decreased the levels of TGF-β1 receptor II, altogether inhibiting the activation of Smad2 and Smad3[48]. Considering the significance of the TGF-β1/Smad signaling pathway in regulating fibrogenesis, we are trying to detect the change of TGF-β1 and Smad3 in rats from different group. The data obtained from HF-treated rats revealed that HF administration attenuated level of the prominent profibrogenic cytokine TGF-β1 (Figure 6B) indicating the inhibitory activity of HF to the proliferative activity of HSCs which might be confirmed by the less collagen deposition in the liver tissues of animals treated with HF ( Figure 4). Similarly, the high levels of Smad3 (Figure 6A) observed in the fibrosis group rats were reversed in the rats treated with HF. Therefore, the decreased fibrosis level after HF treatment may be mediated via its inhibition of the TGF-β1/Smad3 signaling pathway.

Since ConA-induced liver fibrosis is caused by the repeated activation of T cells, the potential immunomodulatory effect contained in the HF. Treatment with HF resulted in inhibition of NF-kB which plays a critical role in the regulation of immunity and inflammation by stimulating the transcription of a wide range of cytokine-encoding genes, including TNF-α and IFN-γ[49]. Indeed, orally administration of HF significantly suppressed the levels of secreted pro-inflammatory cytokines TNF-α, IL-1β and IL-6(Figure 7), elicited in vivo by the T cell mitogen, ConA. These cytokines are known to regulate collagen synthesis[29], and thus, are likely to be involved in the anti-fibrotic effect of halofuginone. In addition to its ability to regulate cytokine production, NF-kB is involved in regulation of the acute-phase response of inflammation, which provides systemic defense and restores homeostasis after infection or injury [50,51]. Thus, HF appears to act anti-inflammatory properties in the process of inflammation, this may be another reason for HF on liver fibrosis.

In conclusion, the present study has for the first time systemically investigated the potential protective role of HF on ConA-induced liver fibrosis models, the prophylactic effect of HF on long-term ConA-induced liver fibrosis including anti-fibrosis and anti-inflammation. The results indicate that targeting HF may present a potent approach, particularly for its prophylactic effects, against liver fibrosis. Preclinical trials by applying some promising HF have already been launched though at their infancy. Although the characteristics of a putative HF receptor and the exact downstream signaling pathways are still obscure, our findings provide additional information toward elucidating its mode of action and therapeutic potential.
